# Immunocytochemical Analysis of Endogenous Frizzled-(Co-)Receptor Interactions and Rapid Wnt Pathway Activation in Mammalian Cells

**DOI:** 10.3390/ijms222112057

**Published:** 2021-11-08

**Authors:** Jochen Neuhaus, Annett Weimann, Mandy Berndt-Paetz

**Affiliations:** Department of Urology, Research Laboratories, University of Leipzig, 04109 Leipzig, Germany; Jochen.Neuhaus@medizin.uni-leipzig.de (J.N.); Annett.Weimann@medizin.uni-leipzig.de (A.W.)

**Keywords:** Wnt signaling, receptors, interaction, proximity ligation assay, PC-3 cells

## Abstract

The differential activation of Wnt pathways (canonical: Wnt/β-catenin; non-canonical: planar cell polarity (PCP), Wnt/Ca^2+^) depends on the cell-specific availability and regulation of Wnt receptors, called Frizzled (FZD). FZDs selectively recruit co-receptors to activate various downstream effectors. We established a proximity ligation assay (PLA) for the detection of endogenous FZD–co-receptor interactions and analyzed time-dependent Wnt pathway activation in cultured cells. Prostate cancer cells (PC-3) stimulated by Wnt ligands (Wnt5A, Wnt10B) were analyzed by Cy3-PLA for the co-localization of FZD6 and co-receptors (canonical: LRP6, non-canonical: ROR1) at the single-cell level. Downstream effector activation was assayed by immunocytochemistry. PLA allowed the specific (siRNA-verified) detection of FZD6–LRP6 and FZD6–ROR1 complexes as highly fluorescent spots. Incubation with Wnt10B led to increased FZD6–LRP6 interactions after 2 to 4 min and resulted in nuclear accumulation of β-catenin within 5 min. Wnt5A stimulation resulted in a higher number of FZD6–ROR1 complexes after 2 min. Elevated levels of phosphorylated myosin phosphatase target 1 suggested subsequent Wnt/PCP activation in PC-3. This is the first study demonstrating time-dependent interactions of endogenous Wnt (co-)receptors followed by rapid Wnt/β-catenin and Wnt/PCP activation in PC-3. In conclusion, the PLA could uncover novel signatures of Wnt receptor activation in mammalian cells and may provide new insights into involved signaling routes.

## 1. Introduction

Wnt (*Wingless and Int1*) signaling pathways play essential roles in embryonic development as well as in the maintenance of tissue homeostasis in adults by regulating proliferation, migration, and differentiation. Dysfunction of Wnt signaling is observed in several human pathologies including cancer [1,2,3]. Wnt proteins bind to seven-transmembrane receptors, the so-called Frizzled (FZD) receptors [4]. FZDs can act together with a variety of co-receptors including low-density lipoprotein receptor (LRP) 5 and LRP6, inactive tyrosine-protein kinase transmembrane receptor (ROR) 1 and ROR2, and tyrosine-protein kinase RYK to activate canonical (β-catenin-dependent) and non-canonical (β-catenin-independent) signaling pathways [3,5]. The canonical Wnt/β-catenin pathway is initiated by the formation of a complex between Wnt, FZD, and LRP5/6, resulting in subsequent stabilization and nuclear translocation of the transcriptional regulator β-catenin [6]. In contrast, mammalian β-catenin-independent Wnt pathways are more diverse but can be sub-divided into planar cell polarity (PCP) and the Wnt/Ca^2+^ signaling pathways. The Wnt/PCP pathway is initiated by interactions of FZDs with ROR co-receptors. Further, PCP signaling involves the small GTPase Rho, which activates the Rho-associated kinase cascade, and the GTPase Rac, which is associated with signaling via JNK and AP1 transcription factors. Subsequent phosphorylation of myosin light-chain phosphatase and dynamic cytoskeletal rearrangements, as well as transcriptional changes, regulate cell polarity, adhesion, and migration [7]. Activation of non-canonical Wnt/Ca^2+^ signaling stimulates the mobilization of free intracellular Ca^2+^ and activates G proteins, protein kinase C, and calcium/ calmodulin-dependent kinase II, which mediate actin polymerization [7,8]. Although different Wnt signaling pathways have been characterized, the underlying mechanisms by which Wnt proteins activate certain signaling routes are poorly understood [9]. There is emerging evidence that distinct FZDs selectively recruit co-receptors in specific cellular contexts, thereby influencing the signaling outcome [10].

While Wnt proteins are best known for their role during embryonic development, Wnt signaling has also been linked to different types of cancer, including prostate cancer (PCa). In this regard, most attention has been focused on the activity of the Wnt/β-catenin pathway. Many prostate tumors have increased levels of β-catenin in the cytoplasm and/or nucleus resulting from gene mutations or non-genomic alterations in the expression of Wnt pathway inhibitors and activators [11]. Only few studies have addressed the significance of non-canonical Wnt signaling via upregulated Wnt5A in PCa [12,13]. Signal transduction of Wnt5A and other Wnt ligands largely depends on the cell-specific availability of Wnt receptors [14]. Several FZDs and ROR1 were shown to be upregulated in human PCa [15,16,17,18,19]. A newly discovered non-canonical Wnt signaling route, via Wnt5A–FZD2 interaction, was shown to be associated with epithelial-to-mesenchymal transition in PCa [15]. In addition, Wnt5A promoted tumor cell invasion through FZD2–ROR2 interaction in PCa cell lines [12]. Another study explored the tumorigenic effects of Wnt5A/FZD5 and Wnt5A/RYK signaling in transfected PCa cells [20]. Hence, cultured PCa cells represent an appropriate model to investigate canonical as well as non-canonical Wnt receptor regulation.

However, no short-term interactions between endogenous FZDs and co-receptors were studied in situ. In the present report, we established a proximity ligation assay (PLA) to detect and quantify the co-localization of FZD6 with the major canonical co-receptor LRP6 as an early event of the well-documented Wnt/β-catenin signal transduction after Wnt10B stimulation in PC-3 prostate cancer cells. In addition, we verified PLA specificity for the detection of FZD6 receptor complexes by siRNA-mediated FZD6 knockdown. To explore non-canonical receptor action in PC-3, we additionally analyzed interactions between FZD6 and ROR1 (exclusively non-canonical action) after Wnt5A treatment. Indications of subsequent Wnt/PCP activation were studied by immunofluorescence-based detection of phosphorylated myosin phosphatase target subunit 1 (phospho-MYPT1), a Wnt/PCP effector downstream of Rho. The results of the Wnt/PCP pathway in PC-3 cells were comparatively analyzed in HEK293 cells.

## 2. Results

### 2.1. Detection of Wnt (Co-)Receptor Interactions

**Figure 1:**
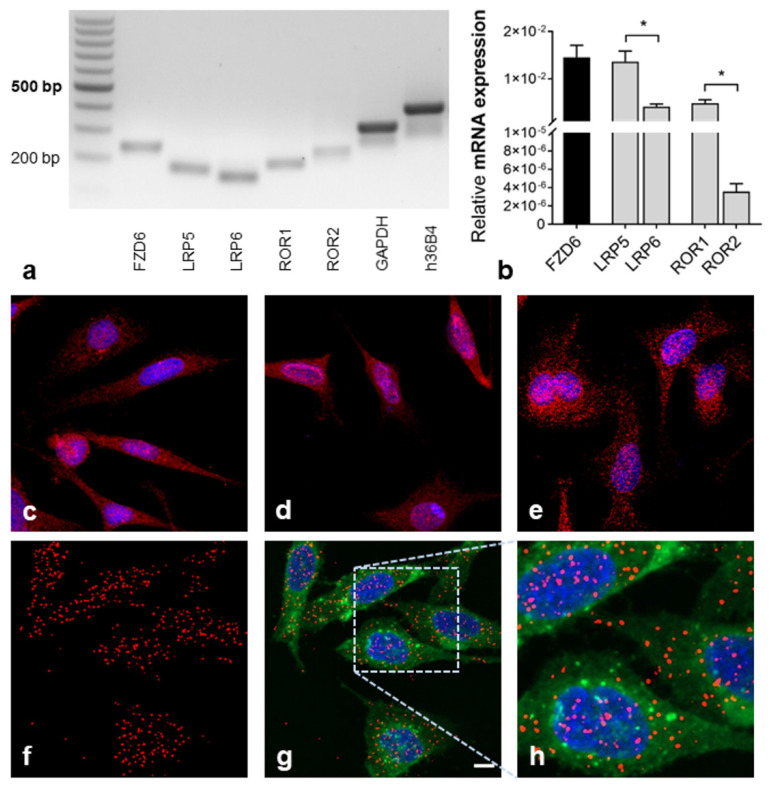
Wnt (co-)receptor expression and interactions. (**a**,**b**) Wnt (co-)receptor expression by qRT-PCR. (**a**) Qualitative presentation of the expression; (**b**) Relative mRNA expression of target genes was quantified (normalized to h36B4 expression). Quantification revealed high expression of FZD6, LRP5/6, and ROR1, while ROR2 was almost not expressed (* *p* ≤ 0.05, Mann–Whitney Test); mean + SD; *n* = 3 independent experiments. (**c**–**e**) Immunofluorescence staining of FZD6 (**c**), LRP6 (**d**), and ROR1 (**e**). Receptors (red); nuclei (blue). (**f**–**h**) Visualization of the co-localization between FZD6 and co-receptors by PLA. Prominent fluorescent spots indicate receptor interactions, exemplarily shown for FZD6–LRP6, (**f**) Cy3-PLA for FZD6–LRP6; (**g**,**h**) merged images of Cy3-PLA, membrane labeling by Alexa Fluor 488^®^-coupled wheat-germ agglutinin (WGA) and nuclei staining with DAPI, (**d**,**e**) merged images. FZD6–LRP6 complexes (red); plasma membranes (green); nuclei (blue). Scale bar: 10 µm.

Co-localization of Wnt receptors (FZD) and co-receptors was mentioned to be the first event in Wnt signal transduction [3,5]. We established a PLA to detect and quantify the co-localization of FZD6 and co-receptors in PC-3 cells, which indicates an interaction between these molecules. Quantitative RT-PCR revealed mRNA expression of FZD6, LRP5/6, and ROR1, with very weak ROR2 expression (Figure 1a,b). Since LRP6 was reported to be the major co-receptor in Wnt/β-catenin signaling, FZD6 interactions with LRP6 were analyzed to study the canonical Wnt route. For Wnt/PCP, we determined FZD6–ROR1 interactions in PC-3 cells. Immunofluorescence staining confirmed the abundance of FZD6, LRP6, and ROR1 at the protein level in PC-3 cells (Figure 1c–e). Antibodies were previously tested in positive control tissue (including technical negative controls; Figure S1a) to demonstrate target specificity in situ. Additionally, antibodies were applied in positive control cells to show their suitability for immunofluorescence (Figure S1b). The application of primary antibodies against FZD6 and co-receptors in PLA allowed the visualization of FZD6 co-localized with LRP6, as well as the detection of FZD6 co-localized with ROR1 receptors. Representative images of highly fluorescent red dots showing FZD6–LRP6 interaction complexes are supplied in Figure 1f–h. Negative control staining was performed by incubation with control IgG followed by treatment with PLA probes. The controls were almost free of PLA signals (Figure S2).

**Figure 2:**
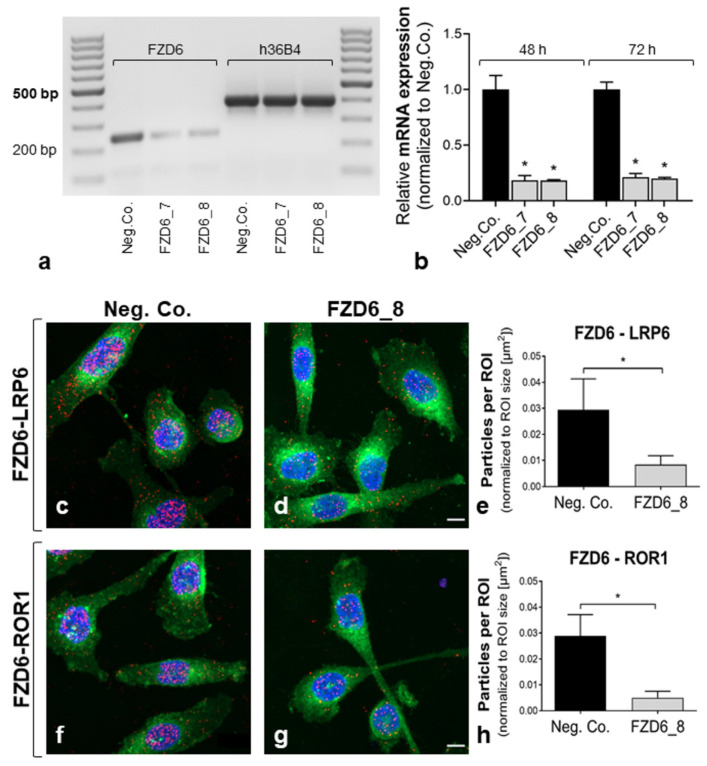
Verification of PLA specificity for the detection of FZD6 receptor complexes. Analysis of FZD6 receptor complexes after siRNA-mediated knockdown of FZD6. (**a**,**b**) *FZD6* expression by qRT-PCR after treatment with specific siRNAs (FZD6_7, FZD6_8; vs. Neg. Co. siRNA). (**b**) The relative mRNA expression of *FZD6* (normalized to *h36B4* expression) was quantified 48 and 72 h after siRNA treatment. Quantification revealed a significant *FZD6* knockdown (75%–80%) by siRNA targeting FZD6 vs. non-targeting siRNA (Neg. Co.). (**c**–**h**) Co-localization of FZD6 with co-receptors LRP6 and ROR1 by PLA after treatment with FZD6_8 siRNA for 48 h. FZD6–LRP6/ROR1 complexes (red); plasma membranes (green); nuclei (blue). Scale bar: 10 µm. (**e**,**h**) The quantification of PLA signal density was performed by particle analyses at the single-cell level; particles were normalized to the area of cells. The number of FZD6–LRP6 and FZD6–ROR1 complexes was significantly decreased (70%–80%) after siRNA-mediated FZD6 knockdown (in accordance with mRNA expression after siRNA). * *p* ≤ 0.05, Mann–Whitney Test; mean + SD; *n* = 3 independent experiments.

The specificity of PLA for the detection of FZD6–LRP6 and FZD6–ROR1 complexes was verified in PC-3 after siRNA-mediated FZD6 knockdown (Figure 2). We used two FZD6-specific siRNAs (FZD6_7, FZD6_8) versus non-targeting siRNA (Neg. Co.) for the initial experiments. Quantification of mRNA expression revealed a significant FZD6 knockdown by FZD6_7 and FZD6_8 (79%-82%) after 48 to 72 h of incubation (Figure 2a,b). PLA was performed 48 h after transfection with FZD6_8 siRNA. Signals for FZD6–LRP6 and FZD6–ROR1 complexes were detected and quantified by particle analyses in WGA-positive cell areas at the single-cell level. In accordance with the reduced FZD6 mRNA levels, the amounts of FZD6–LRP6 (71.3% vs. Neg. Co.) and FZD6–ROR1 (82.7% vs. Neg. Co.) complexes were significantly decreased (*p* < 0.0001, Mann–Whitney test) after siRNA-mediated FZD6 knockdown (Figure 2c–h).

### 2.2. Rapid Activation of the Pathway

**Figure 3:**
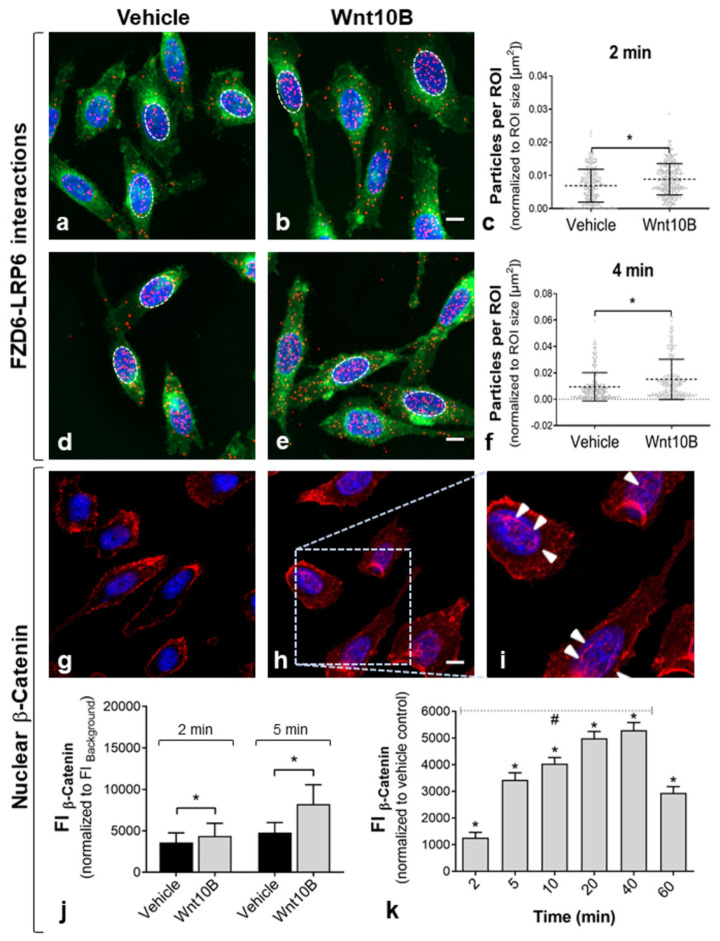
Wnt10B-dependent activation of the canonical Wnt/β-catenin pathway following FZD6–LRP6 co-localization. Co-localization of FZD6 and LRP6 by Wnt10B treatment and subsequent detection of nuclear β-catenin indicates rapid receptor interaction and early Wnt/β-catenin signaling in PC-3. (**a**–**f**) Detection of FZD6–LRP6 interaction complexes by PLA after 2 and 4 min of Wnt10B incubation (**b**,**e**) versus vehicle-treated controls (**a**,**d**). FZD6–LRP6 interaction complexes were frequently observed in the nuclei (circles). FZD6–LRP6 complexes (red); plasma membranes (green); nuclei (blue). Scale bar: 10 µm. (**c**,**f**) Quantification of PLA signal density by particle analyses at the single-cell level. Particles were normalized to the area of cells (ROIs). The amount of FZD6–LRP6 complexes was significantly higher after both 2 min (*n* = 317 vs. *n* = 297 cells) and 4 min (*n* = 264 vs. *n* = 263 cells, vehicle vs. Wnt10B) of Wnt10B incubation (* *p* ≤ 0.05, Mann–Whitney Test); mean + SD; *n* = 3 independent experiments. (**g**–**k**) Well-documented translocation of β-catenin into the nucleus. (**g**–**i**) Immunofluorescence images of β-catenin staining. (**g**) Membrane association of β-catenin in control cells; (**h**,**i**) nuclear localization of β-catenin after incubation of PC-3 cells for 20 min with Wnt10B; arrows indicate nuclear β-catenin labeling. β-Catenin (red); nuclei (blue). Scale bar: 10 µm. (**j**,**k**) Quantification of β-catenin (FI, fluorescence intensity) in DAPI-positive nuclei. Significantly increased nuclear β-catenin was detected within 2 to 5 min of Wnt10B incubation (**j**) and continued until 60 min of incubation (**k**); * significance vs. vehicle-treated control (* *p* ≤ 0.05, Mann–Whitney Test); (**k**) nuclear β-catenin (normalized to vehicle-treated control) followed a linear trend until 40 min of Wnt10B incubation (# *p* ≤ 0.05, one-way ANOVA, post hoc test for linear trend, *R*2 = 0.915); mean + SD; *n* = 3 independent experiments.

Induction of the Wnt/β-catenin pathway was provoked by treatment with Wnt10B, a Wnt ligand associated with canonical Wnt signaling [21]. Co-localization of FZD6 and the major canonical co-receptors LRP6 was analyzed by PLA after short-term Wnt10B incubation (Figure 3). Treatment with Wnt10B led to a rapid increase of PLA signals in PC-3, indicating enhanced interactions of FZD6 and LRP6 (Figure 3a–f). The number of FZD6-LRP6 complexes was significantly higher already after 2 min of Wnt10B incubation (Figure 3c). The significant increase in FZD6–LRP6 complexes continued until 4 min in Wnt10B-treated PC-3 cells versus controls (Figure 3f). Interestingly, the number of FZD6-LRP6 complexes was not increased after 10 min of Wnt10B incubation and was even significantly reduced after 30 min (Figure S3). Based on these results, FZD6 receptor interactions were analyzed after 2 and 4 min in the present study. In addition, FZD6–LRP6 complexes were mainly observed in the nucleus of PC-3 after 2 and 4 min of treatment (Figure 3a–d). Moreover, when using the non-canonical ligand Wnt5A for the stimulation, the number of FZD6–LRP6 interactions was significantly reduced after 2 min of incubation (Figure S4a).

Nuclear translocation of β-catenin was analyzed using immunofluorescence to examine the subsequent downstream activation of the canonical Wnt/β-catenin pathway. Wnt10B incubation resulted in an obvious nuclear accumulation of β-catenin in PC-3 cells (Figure 3g–i). Fluorescence intensity of β-catenin staining was quantified in DAPI-positive nuclei. Significantly enhanced nuclear β-catenin was already detected within 2 to 5 min of Wnt10B incubation (Figure 3j). The increase of nuclear β-catenin (normalized to vehicle-treated controls) followed a significant linear trend until 40 min of Wnt10B treatment (*p* < 0.0001, one-way ANOVA, post hoc test for linear trend). There was a decrease in nuclear β-catenin in Wnt10B-treated cells vs. controls after 60 min (Figure 3k).

### 2.3. Activation of Non-Canonical Wnt/PCP Signaling

**Figure 4:**
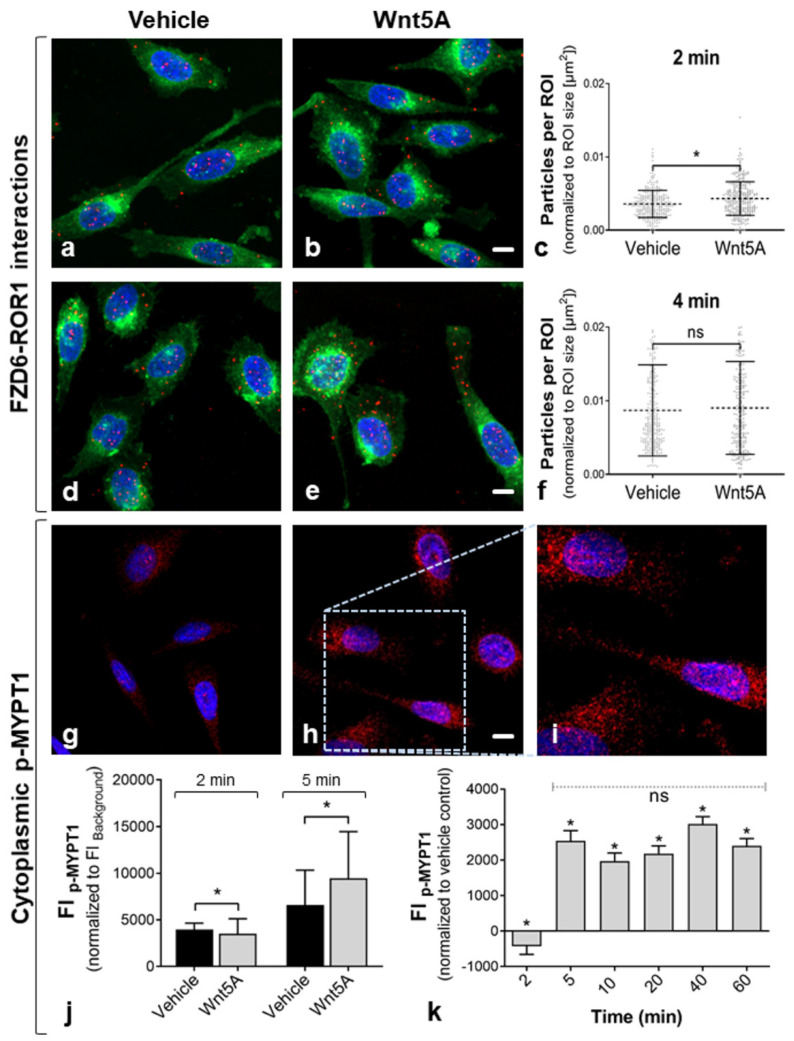
Wnt5A-dependent activation of the non-canonical Wnt/PCP pathway by Wnt5A following short-term co-localization of FZD6–ROR1. Co-localization of FZD6 and ROR1 by Wnt5A treatment and subsequent detection of cytoplasmatic p-MYPT1 suggests a rapid receptor interaction and early Wnt/PCP signaling in PC-3. (**a**–**h**) FZD6–ROR1 interaction complexes in PC-3 cells after 2 and 4 min of Wnt5A incubation (**b**,**e**) versus vehicle-treated controls (**a**,**d**). FZD6–ROR1 complexes (red); plasma membranes (green); nuclei (blue). Scale bar: 10 µm. (**c**,**f**) Quantification of PLA signal densities by particle analyses at the single-cell level. Particles were normalized to the area of cells (ROIs). The amounts of FZD6–ROR1 complexes was higher after 2 min (*n* = 306 vs. *n* = 304 cells) of incubation with Wnt5A, while there was no difference after 4 min (*n* = 264 vs. *n* = 263 cells, vehicle vs. Wnt5A; * *p* ≤ 0.05, Mann–Whitney Test); mean + SD; *n* = 3 independent experiments. (**g**–**k**) Increase of p-MYPT1 (Thr696) in the cytoplasm. (**g**–**i**) Immunofluorescence images of p-MYPT1 staining. (**g**) Low levels of p-MYPT1 in control cells; (**h**,**i**) increased levels of p-MYPT1 after incubation of PC-3 cells for 5 min with Wnt5A. p-MYPT1 (red); nuclei (blue). Scale bar: 10 µm. (**j**,**k**) Quantification of p-MYPT1 (FI, fluorescence intensity) in the cytoplasm. Significantly increased cellular p-MYPT1 was detected after 5 min and up to 60 min of Wnt5A incubation; * significance vs. vehicle-treated control (* *p* ≤ 0.05, Mann–Whitney Test); (**k**) cellular p-MYPT1 levels (normalized to vehicle-treated control) remained constant until 60 min of Wnt5A treatment. (ns = not significant, *p* ≤ 0.05, one-way ANOVA, post hoc test for linear trend, *R*2 = 0.109); mean + SD; *n* = 3 independent experiments.

Treatment with Wnt5A has been shown to activate the non-canonical Wnt/PCP pathway [12,13]. To investigate non-canonical Wnt/PCP signaling in PC-3 cells, we performed a PLA for FZD6 and its non-canonical co-receptor at 2 and 4 min of Wnt5A incubation (Figure 4). Since *ROR2* was weakly expressed at the mRNA level and Wnt5A stimulation had no effect on the amount of FZD6–ROR2 interaction complexes by PLA (data not shown), only FZD6–ROR1 interactions were analyzed further. Treatment with Wnt5A for 2 min resulted in significantly increased levels of FZD6–ROR1 complexes, while there was no difference after 4 min of Wnt5A incubation (Figure 4c,f). In addition, FZD6–ROR1 complexes were distributed throughout the cell/cytoplasm (Figure 4a,b,d,e). In turn, treatment with the canonical Wnt10B ligand showed no significant effect on FZD6–ROR1 levels in PC-3 (Figure S4b). Indication of Wnt/PCP downstream activation was analyzed by determining the cellular levels of the phosphorylated form of MYPT1 (protein phosphatase 1 regulatory subunit 12A, p-MYPT1, Thr696), a Wnt/PCP target protein, using quantitative immunofluorescence (Figure 4g–i). Fluorescence intensity of p-MYPT1 staining was quantified in WGA-stained cells. Significantly elevated levels of cellular p-MYPT1 were detected after 5 min but not after 2 min of Wnt5A incubation (Figure 4j). After 5 min, the level of cellular p-MYPT1 (normalized to vehicle-treated controls) remained constant until 60 min of Wnt5A stimulation (Figure 4k). Since Wnt/PCP was shown to play a major role during embryonic kidney development in mammals [22], we verified these findings in the well-established human embryonic kidney cell line HEK293 as a Wnt/PCP-positive control. The results of this verification are summarized in Supplemental Figure S5. Significantly higher amounts of FZD6–ROR1 and FZD6–ROR2 complexes were detected after treatment of HEK293 with Wnt5A (Figure S5a–f). In addition, Wnt5A treatment led to significantly increased levels of cellular p-MYPT1 after 5, 10, and 20 min (Figure S5g–i).

## 3. Discussion

Wnt signaling is categorized into canonical, β-catenin-dependent and non-canonical, β-catenin-independent pathways [1]. The categorization is not absolute, as Wnt signaling is a dynamic and complex regulatory pathway and signaling routes show some cross talks [14]. Wnt pathway activity can be positively or negatively regulated by various protein–protein interactions in response to the dynamic cellular environment [23]. In this context, the coupling selectivity of activated FZDs to downstream signaling pathways via selective recruitment and participation of co-receptors can influence the signaling outcome. However, the specificity of FZD–co-receptor interactions remains unresolved [10]. In the present study, we addressed three issues regarding Wnt signaling in mammalian cells: (i) whether we are able to detect and quantify endogenous interactions of FZDs and co-receptors after Wnt ligand treatment, (ii) whether we can subsequently observe time-dependent activation of downstream effectors, and (iii) whether unknown Wnt signaling routes exist in the used cell model.

Wnt signaling is recognized as an important contributor to PCa progression [11], and activation of Wnt signaling by multiple Wnt ligands has been shown in several in vitro studies using PCa cell lines [12,24,25]. Thus, PC-3 prostate cancer cells were used for the experiments. Using the novel proximity ligation assay (PLA), we here investigated the co-localization of FZD6 and the co-receptors LRP6 and ROR1, as an early event of Wnt signal transduction after treatment with Wnt ligands. FZD6 was found to be upregulated in human PCa samples, while the effect on Wnt signaling is unknown [18]. In PC-3 cells, FZD6 showed solid abundance as determined by quantitative RT-PCR and immunofluorescence before performing the PLA experiments. PLA signals representing FZD6 interactions with LRP6 and ROR1 were visible as fluorescent red dots (Figure 1) and could be detected by particle analysis at the single-cell level. Increased FZD6 interaction complexes with LRP6, the major candidate of the canonical co-receptors, already after 2 min demonstrated immediate release of canonical Wnt/β-catenin signaling in PC-3 cells by Wnt10B stimulation. After 10 and 30 min, the number of FZD6–LRP6 complexes was not significantly increased by Wnt10B (Figure S3). Our results suggest that the interactions with Wnt (co-)receptors are short-term events leading to rapid downstream activation. This is similar to what found by another study showing that Dishevelled (DVL; cytoplasmic phosphoproteins downstream of FZDs) formed membrane-bound oligomers during Wnt pathway activation within 5 min of Wnt3A treatment. DVL complex formation gradually declined over time and was followed by a measurable increase in cytosolic β-catenin after 30 min [26]. In the present study, co-localization of FZD6 and LRP6 was not provoked by the non-canonical ligand Wnt5A (Figure S4), indicating that our approach can be used to study the selective recruitment of FZDs and co-receptors by specific Wnt ligands. Interestingly, FZD6–LRP6 complexes were mainly observed in the nucleus of PC-3 (Figure 3). It has been shown that the soluble intracellular domains of surface receptors, such as FZDs and LRP6, can translocate to the cytoplasm and nucleus, where they could affect gene transcription in a β-catenin-dependent or -independent manner [27,28]. Consistent with the early detection of FZD6–LRP interactions, nuclear translocation of the downstream effector β-catenin was determined even within 5 min and increased linearly until 40 min (Figure 3). Other studies analyzed nuclear β-catenin translocation not earlier than after 20 to 30 min [25,29]. Our results for the first time demonstrated Wnt pathway activation at the FZD/LRP level, followed by rapid β-catenin signaling and give new insights into the dynamics of an otherwise well-described Wnt signaling route in PC-3 cells.

Although FZD6 has been shown to regulate both canonical and non-canonical Wnt pathways, most reports indicate a prevalent role in non-canonical Wnt signaling [30,31]. In PC-3 cells, activation of Wnt/Ca^2+^ signaling via G proteins has been well documented as a major pathway of the non-canonical signaling to regulate cell motility in PCa [25,32]. However, an emerging theme is the activation of other non-canonical pathways by specific Wnt5A/FZD–co-receptor complexes in PCa. For instance, increased activation of non-canonical Wnt pathways through Wnt5A–FZD2 has been detected as a novel mode of Wnt activation in PCa tissue [15]. Yamamoto et al. suggested that Wnt5A promotes migration and invasion through FZD2 and ROR2 in cultured PC-3 cells [12]. However, we observed low ROR2 expression in PC-3 compared to ROR1 (Figure 1). This is in accordance with other studies showing rare ROR2 expression in PCa cell lines [20] but upregulated ROR1 expression in PCa tissue [33]. Increased formation of FZD6 interaction complexes with ROR1 suggested short-term release of non-canonical Wnt signaling in PC-3 cells after 2 min of Wnt5A stimulation (Figure 4). Wnt5A–ROR interactions have been shown to induce PCP signaling in developing tissues [3]. In order to show the suitability of the detection method, Wnt/PCP-positive embryonic kidney cells were used for a comparative characterization of FZD6–ROR interactions by PLA in the present study. In this regard, HEK293 showed a higher co-localization of FZD6 and ROR1 and FZD6 and ROR2, followed by an increase of p-MYPT1 (Figure S5). As p-MYPT1 regulates the interaction between actin and myosin downstream of Rho during cytoskeletal changes, p-MYPT1 is considered to be an effector of the PCP pathway [34]. Whether the PCP pathway also plays a major role in oncogenic transformation is still a matter of discussion. However, although we could not detect significant higher amounts of FZD6–ROR1 after 4 min of Wnt5A in PC-3, the levels of cellular p-MYPT1 were significantly increased after 5 min of Wnt5A stimulation (Figure 4). To the best knowledge of the authors, this is the first study suggesting active Wnt/PCP signaling in cultured PCa cells. In recent studies, it has been shown that several core components of the PCP pathway are differently expressed in tumors, contributing to cell migration and metastasis formation. In particular, FZD6 is supposed to play a key role in mediating Wnt/PCP signaling in malignancies [35]. A connection between FZD6 and Wnt/PCP has not been described previously in PCa.

Our results support the view that Wnt signaling is a fast, highly dynamic regulatory pathway whose activation is mediated by various protein–protein interactions. We were able to visualize and quantify the co-localization of FZDs and co-receptors indicating interaction and activation of these molecules after treatment with selectively acting Wnt ligands. Taken together, the PLA enables time-dependent analyses of such endogenous interactions in mammalian cells and could bridge the gap between stimulation and downstream effect. Novel signatures of receptor interaction complexes can give new insights into involved signaling routes and may be linked to various diseases including cancer.

## 4. Materials and Methods

### 4.1. Cell Culture

PC-3 prostate cancer cells (ACC-465) and human embryonic kidney cells HEK293 (ACC-305) were obtained from the Leibniz Institute—German collection of microorganisms and cell cultures (DSMZ, Braunschweig, Germany). PC-3 cells were cultured in RPMI-1640/Ham’s F12 mixture (Thermo Fisher Scientific, Dreieich, Germany) supplemented with 10% fetal calf serum (FCS; Biochrom, Berlin, Germany). HEK293 cells were maintained in 90% Dulbecco’s MEM (with 25 mM HEPES) supplemented with 10% FCS. Prior to treatment, cells were cultured on collagen-A-coated (PC-3) or poly-L-lysine-coated (HEK293) 13 mm coverslips for 24 h. The coating solutions were purchased from Biochrom (Berlin, Germany).

### 4.2. Cell Transfection

FZD6 knockdown was performed to verify PLA detection of FZD6 receptor complexes. siRNAs were purchased from Qiagen (Hilden, Germany). For transfection, PC-3 cells were seeded at a density of 10,000 cells/cm^2^. After overnight incubation, cells were treated using Allstars Negative Control siRNA (Neg. Co., #SI03650318) and specific FlexiTube siRNAs targeting FZD6 (FZD6_7, #SI02757657; FZD6_8, #SI02757664). Transfection was conducted using INTERFERin™ (Polyplus, Illkirch, France) according to the manufacturer´s recommendation. FZD6 knockdown was analyzed after 48 and 72 h.

### 4.3. RNA Extraction and qRT-PCR

For analyzing mRNA expression of Wnt (co-)receptors in PC-3, total RNA was isolated with the RNeasy Plus Micro Kit (Qiagen, Hilden, Germany) according to the manufacturer´s manual. Quantitative PCR was done on a Mastercycler ep Realplex II (Eppendorf, Hamburg, Germany) using SYBR-Green quantitative PCR Mastermix (Thermo Fisher Scientific, Dreieich, Germany) and custom primers (MWG-Biotech, Ebersberg, Germany) (Table 1). The constantly expressed human acidic ribosomal phosphoprotein p0 (*h36B4*) served as a reference gene. Comparable efficiencies of primers used for target and housekeeping gene amplification were determined by analyzing serial cDNA dilutions.

### 4.4. Wnt Ligand Treatment

Recombinant Wnt5A and Wnt10B peptides were purchased from R&D Systems (Minneapolis, MN, USA). Wnt peptide stock solutions (100 µg/mL) were prepared in PBS containing 0.1% bovine serum albumin (PBS/0.1% BSA). Cells were treated with Wnt5A or Wnt10B (500 ng/mL in culture medium) for the indicated time periods at 37 °C and assayed for Wnt pathway activation. Co-localization of Wnt (co-) receptors was analyzed after 2 and 4 min of incubation. Downstream effectors of Wnt were assayed after 2 to 60 min. Samples treated with PBS/0.1% BSA diluted in culture medium were used as vehicle-treated controls.

### 4.5. Immunofluorescence

Cells were fixed with ice-cold methanol and permeabilized using a DMSO/Triton-X-100 mixture. Primary antibodies (Table 2) were incubated overnight at 4 °C. Alexa Fluor 555^®^-coupled secondary antibodies (Thermo Fisher Scientific, Dreieich, Germany) diluted in TBS (1 h, room temperature) were used for indirect immunofluorescence of target proteins. Cells were visualized by membrane staining with Alexa Fluor 488^®^-coupled wheat-germ agglutinin (WGA, Cat.-No.: W11261, Thermo Fisher Scientific, Dreieich, Germany). Nuclei were labeled with 4´,6-diamidine-2´-phenylindole dihydrochloride (DAPI, Cat.-No.: D9542, Sigma-Aldrich, Munich, Germany). Samples were analyzed by confocal laser scanning microscopy (LSM 800, Carl Zeiss, Jena, Germany). Fluorescence intensity (553 nm excitation/568 nm emission) of the images was quantified using Fiji, including ImageJ version 1.53 [36].

### 4.6. In Situ PLA

PLA was used to detect endogenous interactions of FZD6 with the Wnt co-receptors LRP6 and ROR1. Cells were fixed with 3.7% formaldehyde for 30 min at 4 °C. Permeabilization and primary antibody treatment were performed as described above. PLA was performed according to the manufacturer´s instructions. Duolink™ In Situ PLA probes (Anti-Rabbit PLUS, Cat.-No.: DUO92002; Anti-Mouse MINUS, Cat.-No.: DUO92004; Anti-Goat MINUS, Cat.-No.: DUO92006; Sigma-Aldrich, Munich, Germany) were applied to the coverslips for 1 h at 37 °C. Following several wash steps, the samples were incubated with the ligation mix for 30 min at 37 °C and with the Cy3 amplification mix (Duolink™ In Situ Detection Reagents Orange, Cat.-No.: DUO92007, Sigma-Aldrich, Munich, Germany) for 100 min at 37 °C. Technical negative control staining was performed by incubation with only one primary antibody, followed by treatment with PLA probe mixture and PLA detection reagents. Alexa Fluor^®^ 488-labeled WGA was used for the visualization of cells, and nuclei were stained with DAPI contained in the Duolink™ In Situ Mounting Medium (Cat.-No.: DUO82040, Sigma-Aldrich, Munich, Germany). A schematic representation of the PLA-based fluorescence staining is given in Supplemental Figure S6. Samples were analyzed with laser scanning microscopy using an LSM 800 (Carl Zeiss, Jena, Germany). Quantification of PLA signals was done by particle analysis using a self-written ImageJ Macro. WGA-positive cells served to define the regions of interest (ROI). An overview of the workflow of particle analysis is given in Figure S7. Approximately 260 to 300 single cells were analyzed per PLA staining and treatment condition. The amounts of particles were normalized to ROI sizes.

### 4.7. Statistical Analysis

Statistical analyses were performed using Prism 8.0 statistical software (GraphPad Software Inc., La Jolla, CA, USA). Bar diagrams present the means + standard deviation from three independent experiments. The Gaussian distribution of values was tested by the D´Agostino–Pearson omnibus normality test. Statistical differences were analyzed by the Mann–Whitney test. Linear trends were analyzed by one-way ANOVA and post hoc test; *p* ≤ 0.05 was considered statistically significant.

## Figures and Tables

**Table 1:** List of primers for the detection of Wnt (co-)receptors by qRT-PCR. Acc.-No., Accession number; bp, base pairs.

Marker	Gene	Acc.-No.	Sequence (5′–3′)	Binding Site	Product Size (bp)
Frizzled class receptor 6	*FZD6*	NM_003506.4	f-CGAATTGGAGTCTTCAGCGG r-TTCCAACCCAGAAGACAGCA	Exon 3 Exon 4	241
LDL receptor-related protein 5	*LRP5*	NM_002335.4	F-TCAGCCCTGGACTTTGATGT R-CCAGTAGAGGTTCTTGCCCA	Exon 9 Exon 10	171
LDL receptor-related protein 6	*LRP6*	NM_002336.3	f-AGAGTCCCAGTTCCAGTGTG r-GCTTTCCAATGCACTGACCA	Exon 18 Exon 19	158
Receptor tyrosine kinase-like orphan receptor 1	*ROR1*	NM_005012.4	F-AATGATGCTCCTGTGGTCCA R-TATCCTGGACTTGCAGTGGG	Exon 3 Exon 4	200
Receptor tyrosine kinase-like orphan receptor 1	*ROR2*	NM_004560.4	F-GCAGCAAGATGGGGATTCTG R-AGCTCCTCCATGAACCTCAC	Exon 8 Exon 9	241

**Table 2:** Overview of the antibodies used for the detection of FZD6–co-receptor interactions and Wnt downstream effectors. Rb, rabbit; Go, goat.

Pathway	Name	Specificity	Host	Source	Dilution
Wnt pathway	FZD6	Frizzled-6 receptor; associated with PCa	Rb	Life Span Biosciences, Seattle, WA	1:200
Canonical Wnt/b-Catenin pathway (receptor)	LRP6	Co-receptor function in Wnt/b-Catenin signaling (major)	Go	Novus Biologicals, Abingdon, UK	1:100
Non-canonical Wnt pathway (receptor)	ROR1	Tyrosine kinase receptor associated with co-receptor function in Wnt/PCP signaling	Go	Abcam, Cambridge, UK	1:100
Canonical Wnt/b-Catenin pathway (effector)	β-Catenin	Total β-Catenin; key downstream activator of canonical Wnt signaling	Rb	Cell Signaling Technology, Frankfurt/Main, Germany	1:100
Non-canonical Wnt pathway (effector)	p-MYPT1	Phospho-Myosin phosphatase target subunit 1; regulates actin–myosin interactions	Go	Acris Antibodies, Herford, Germany	1:200

## Data Availability

The data presented in this study are available on request from the corresponding author.

## Supplementary Materials

The following are available online at https://www.mdpi.com/article/10.3390/ijms222112057/s1.
