# Supplementary material for: Immunocytochemical Analysis of Endogenous Frizzled-(Co-)Receptor Interactions and Rapid Wnt Pathway Activation in Mammalian Cells
Source: Int J Mol Sci. 2021 Nov 8;22(21):12057. doi: 10.3390/ijms222112057 (PMC8584856; doi:10.3390/ijms222112057)
Supplement: Supplementary file 1 [file ijms-22-12057-s001.zip › ijms-1399436-supplementary/Figure S1.pdf]

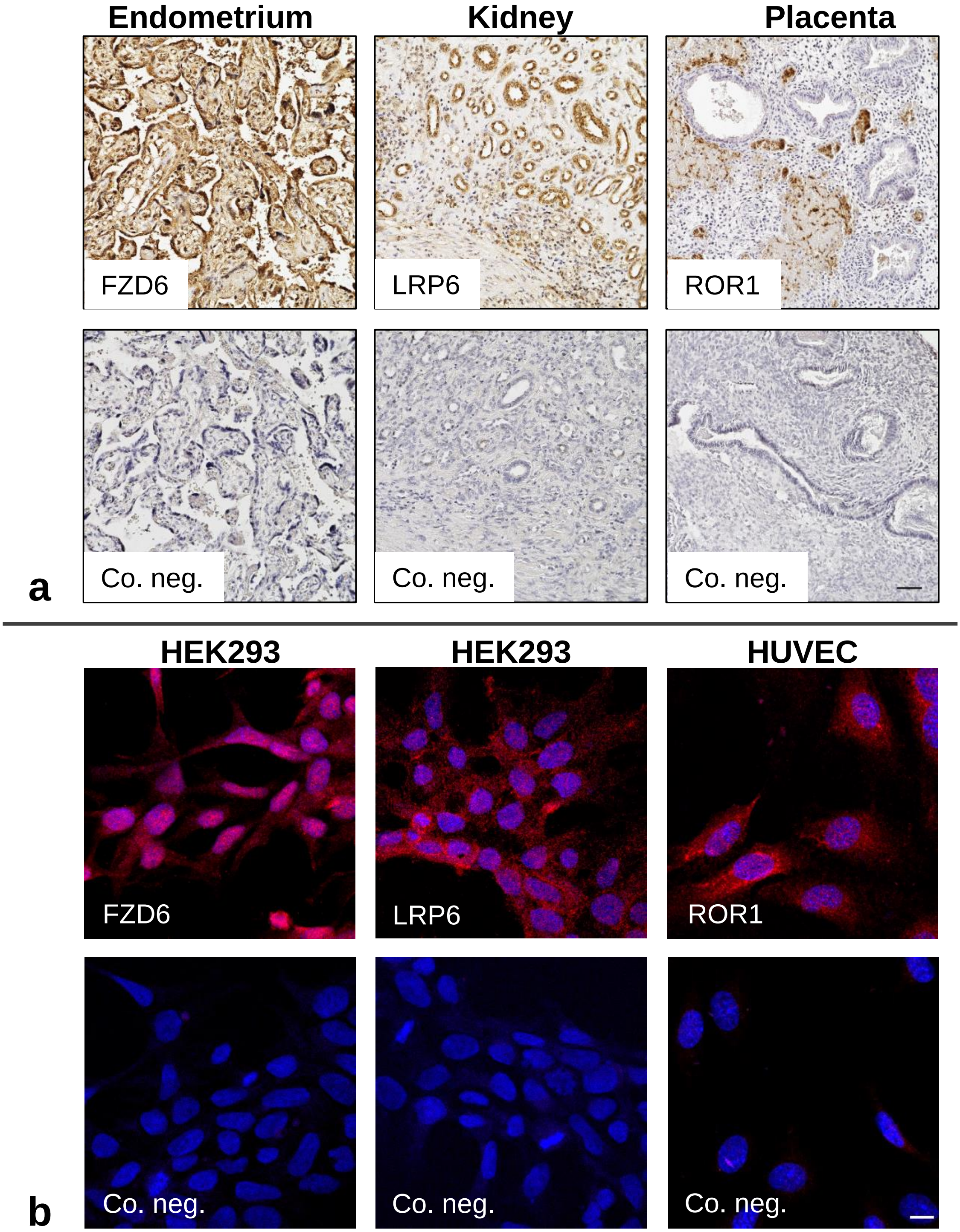

Figure S1: Staining positive controls. (a) Immunhistology of FZD6, LRP6 and ROR1 in positive control tissue (FZD6: human placenta; LRP6: human kidney; ROR1: human endometrium). Formalin-fixed paraffin-embedded tissue was deparaffinized followed by antigen retrieval using heat-induced epitope retrieval buffer (pH 9.0; Zytomed Systems, Berlin, Germany) for 40 mniutes at 100°C. After blocking of endogenous peroxidase, sections were treated with primary antibodies overnight at 4°C. Biotin-linked secondary antibodies were incubated 45 minutes at room temperature. Following a coupling step with horseradish peroxidase streptavidin, DAB was used for visualization and nuclei were counterstained with haematoxylin. Technical negative controls were performed by omitting the primary antibody. Scale bar: 50 µm. (b) Immunfluorescence staining of FZD6, LRP6 and ROR1 in positive control cells (FZD6, LRP6: HEK293; ROR1: HUVEC). Scale bar: 10 µm.
