# Supplementary material for: Immunocytochemical Analysis of Endogenous Frizzled-(Co-)Receptor Interactions and Rapid Wnt Pathway Activation in Mammalian Cells
Source: Int J Mol Sci. 2021 Nov 8;22(21):12057. doi: 10.3390/ijms222112057 (PMC8584856; doi:10.3390/ijms222112057)
Supplement: Supplementary file 1 [file ijms-22-12057-s001.zip › ijms-1399436-supplementary/Figure S2.pdf]

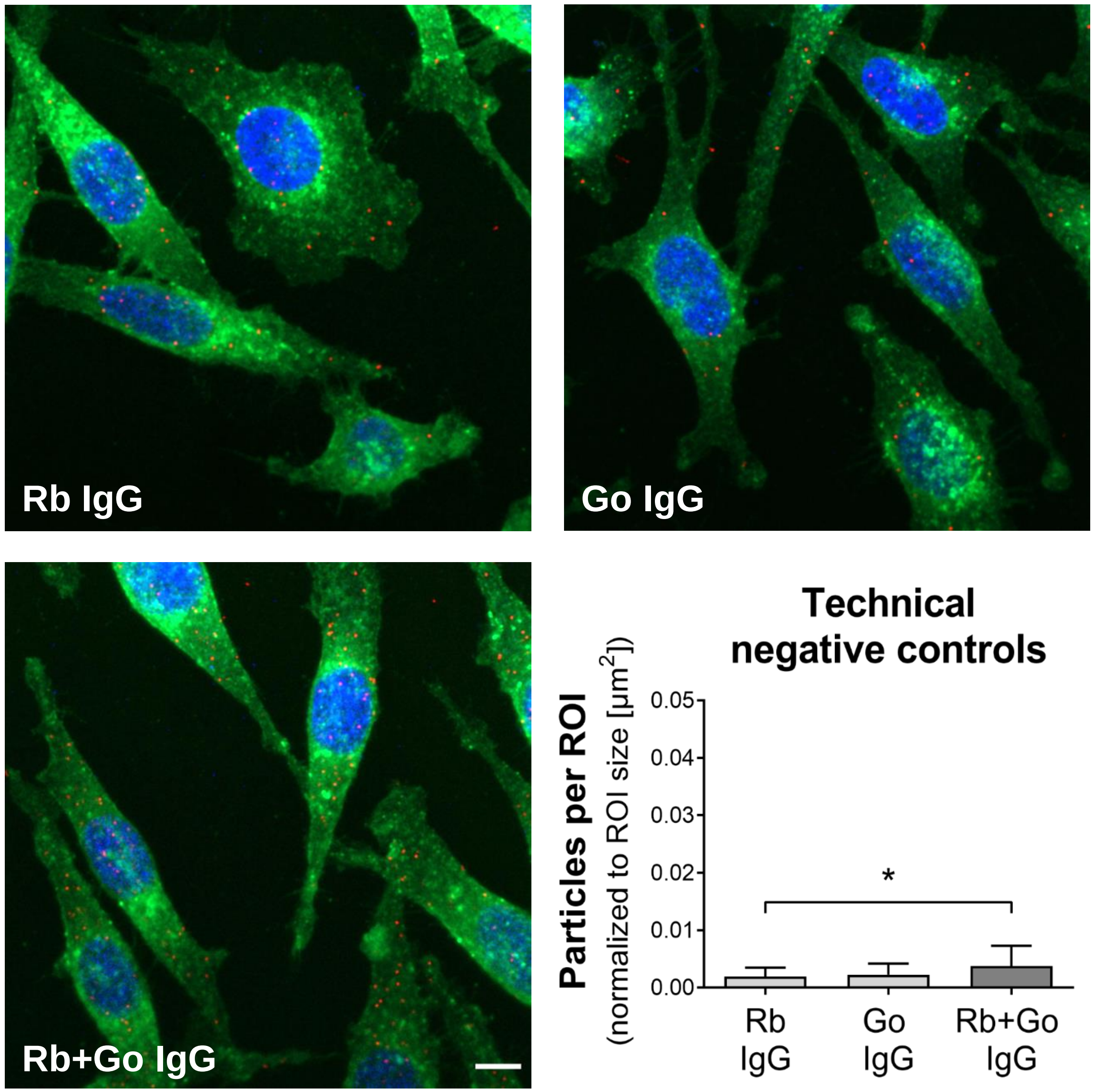

Figure S2: PLA negative controls. Images of technical negative control staining. PLA controls were performed by incubation with rabbit control IgG and/or goat control IgG at the same concentration (5 µg/ml) as the primary antibodies. IgG treatment was followed by incubation with PLA probe mixture. PLA signals were quantified by particle analyses; particles were normalized to the area of the cells (ROIs). Scale bar 10 µm.
