# Supplementary material for: Immunocytochemical Analysis of Endogenous Frizzled-(Co-)Receptor Interactions and Rapid Wnt Pathway Activation in Mammalian Cells
Source: Int J Mol Sci. 2021 Nov 8;22(21):12057. doi: 10.3390/ijms222112057 (PMC8584856; doi:10.3390/ijms222112057)
Supplement: Supplementary file 1 [file ijms-22-12057-s001.zip › ijms-1399436-supplementary/Figure S3.pdf]

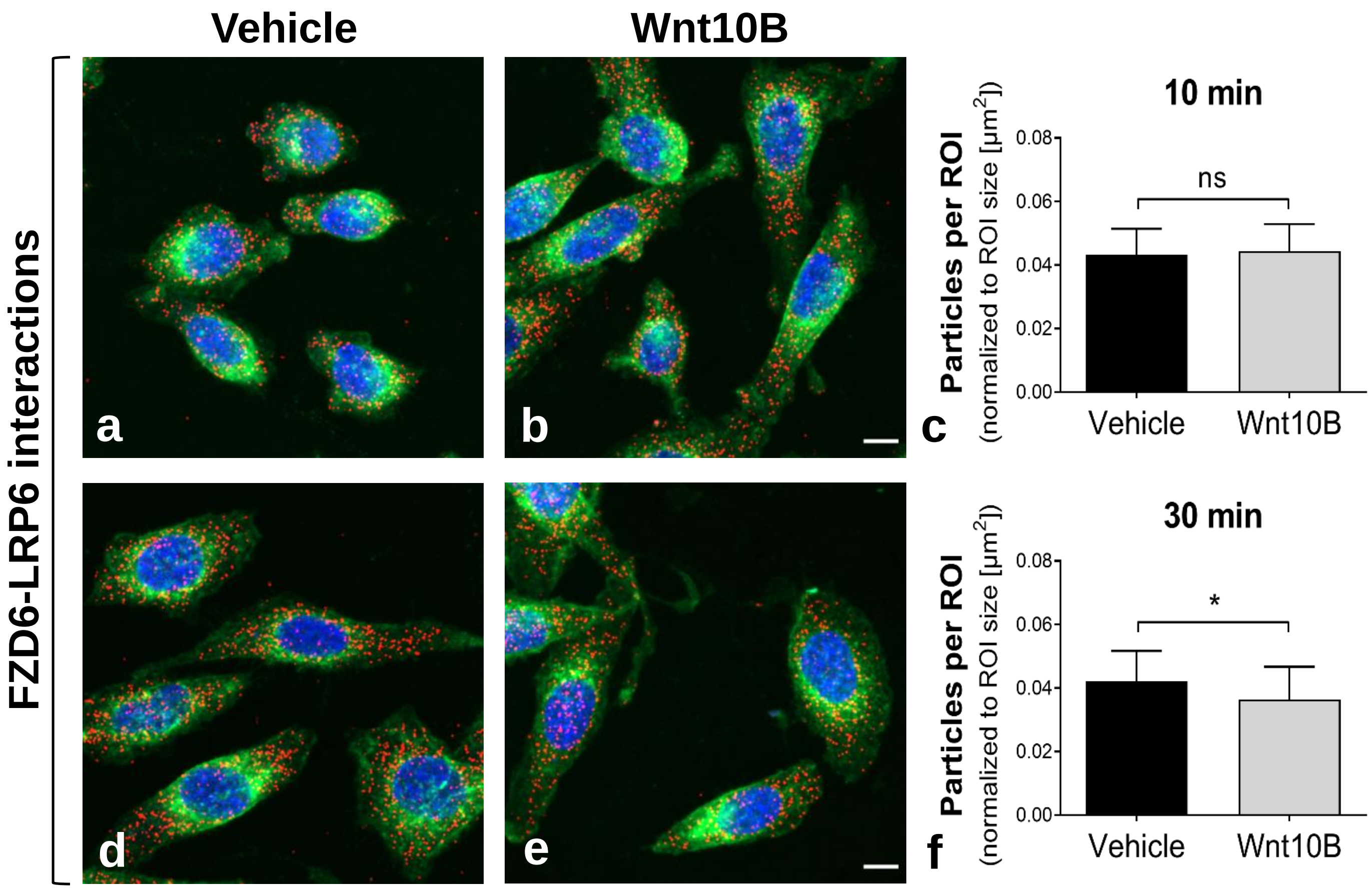

Figure S3: Co-localization of FZD6 and co-receptors by Wnt ligand treatment in a longer time course; exemplarily shown for FZD6-LRP6 interactions by Wnt10B. Detection of FZD6-LRP6 interaction complexes by PLA after 10 and 30 min of Wnt10B incubation (b, e) versus vehicle controls (a, d). FZD6-LRP complexes (red); plasma membranes (green); nuclei (blue). Scale bar: 10 µm. (c, f) Quantification of PLA signal density by particle analyses at single cell level. Particles were normalized to the area of cells (ROIs). Amounts of FZD6-LRP6 complexes were not increased after 10 min of Wnt10B incubation and even reduced after 30 min (* $P \leq 0.05$, Mann-Whitney Test); mean + SD.
