# Supplementary material for: Immunocytochemical Analysis of Endogenous Frizzled-(Co-)Receptor Interactions and Rapid Wnt Pathway Activation in Mammalian Cells
Source: Int J Mol Sci. 2021 Nov 8;22(21):12057. doi: 10.3390/ijms222112057 (PMC8584856; doi:10.3390/ijms222112057)
Supplement: Supplementary file 1 [file ijms-22-12057-s001.zip › ijms-1399436-supplementary/Figure S4.pdf]

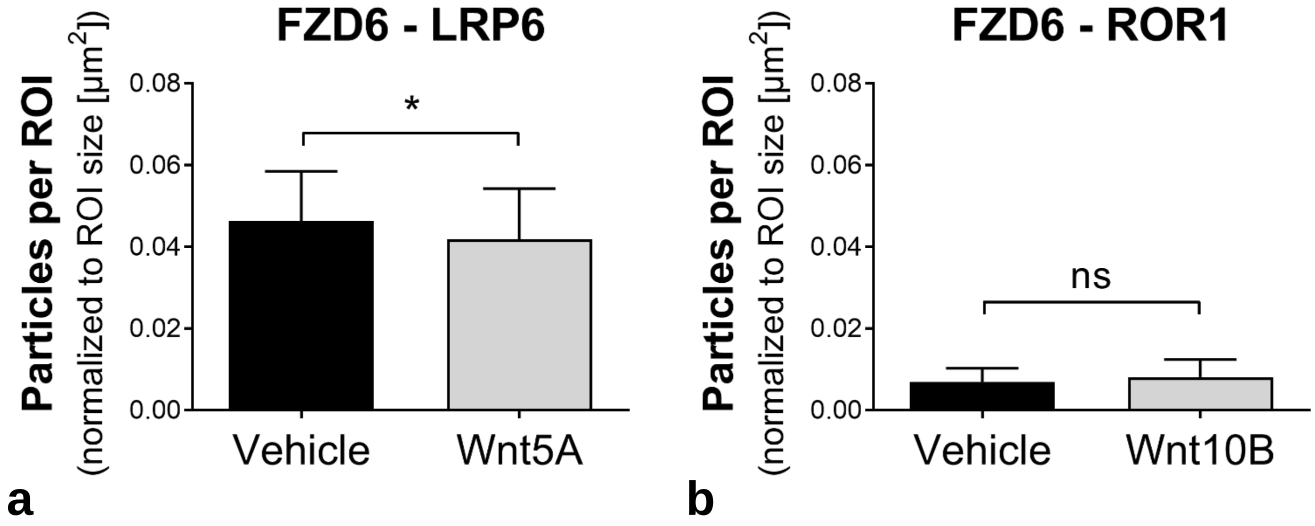

Figure S4: Demonstration of selective FZD (co-) receptor activation by canonical and non-canonical Wnt ligands. Detection of FZD6-LRP6 after 2 min of Wnt5A and FZD6-ROR1 after Wnt10B incubation (versus vehicle controls). Quantification of PLA signal density by particle analyses at single cell level. Particles were normalized to the area of cells (ROIs). (a) Amounts of FZD6-LRP6 complexes were reduced after 2 min incubation with non-canonical Wnt5A ligand. (b) FZD6-ROR1 complexes (non-canonical Wnt) were not significantly different after 2 min of treatment with canonical Wnt10B ligand. (* $P \leq 0.05$, Mann-Whitney Test); mean + SD.
