# Supplementary material for: Immunocytochemical Analysis of Endogenous Frizzled-(Co-)Receptor Interactions and Rapid Wnt Pathway Activation in Mammalian Cells
Source: Int J Mol Sci. 2021 Nov 8;22(21):12057. doi: 10.3390/ijms222112057 (PMC8584856; doi:10.3390/ijms222112057)
Supplement: Supplementary file 1 [file ijms-22-12057-s001.zip › ijms-1399436-supplementary/Figure S5.pdf]

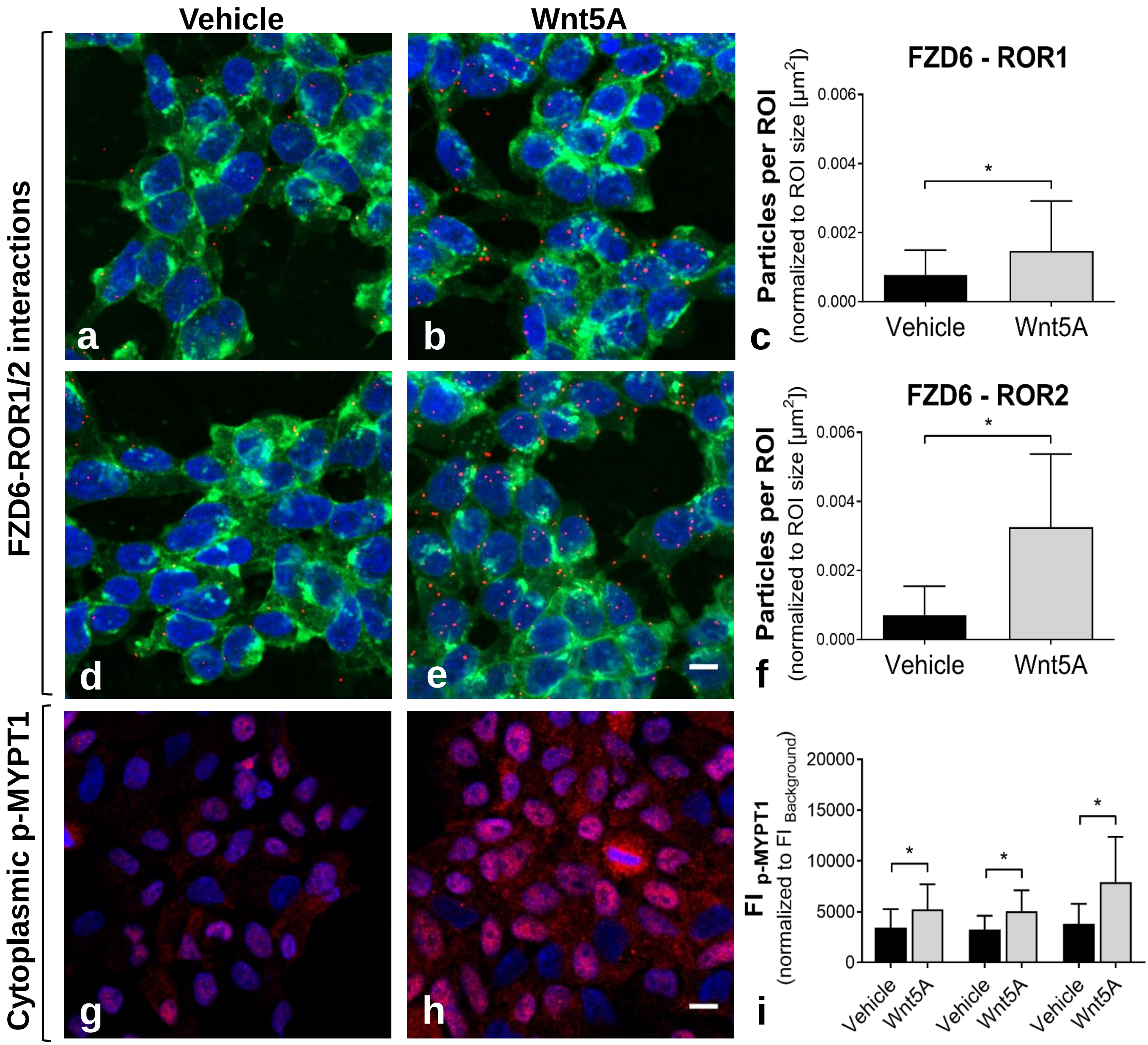

Figure S3: Characterization of the non-canonical Wnt/PCP pathway in HEK293 cells. (a-f) Co-localization of FZD6 and co-receptors ROR1/2 by PLA indicated receptor interaction and early Wnt signal transduction; exemplarily shown for 4 minutes of incubation with Wnt5A. (a, b, d, e) Visualization of high fluorescent spots showing FZD6-ROR1 and FZD6-ROR2 interaction complexes in HEK293 after 4 min of treatment (b, e) versus vehicle controls (a, d). The ROR2 antibody used for PLA was purchased from Thermo Fisher Scientific, Dreieich, Germany (RRID: AB_2725257). FZD6-ROR complexes (red); plasma membranes (green); nuclei (blue). (c, f) Quantification of PLA signal densities by particle analyses. Particles were normalized to the area of cells (ROIs). Amounts of FZD6-ROR1 and FZD6-ROR2 complexes were significantly higher after 4 min incubation with Wnt5A. (g, h) Immunofluorescence images of p-MYPT1 staining; (g) Low levels of p-MYPT1 in control cells; (h) increased levels of p-MYPT1 after incubation of HEK293 for 20 min with Wnt5A. p-MYPT1 (red); nuclei (blue). Scale bar: 10 µm. (i) Quantification of p-MYPT1 (FI, fluorescence intensity) in the cytoplasm; significantly increased cellular p-MYPT1 was detected after 5, 10 and 20 min of Wnt5A incubation (* P ≤ 0.05, Mann-Whitney Test); mean + SD.
