# Supplementary material for: Immunocytochemical Analysis of Endogenous Frizzled-(Co-)Receptor Interactions and Rapid Wnt Pathway Activation in Mammalian Cells
Source: Int J Mol Sci. 2021 Nov 8;22(21):12057. doi: 10.3390/ijms222112057 (PMC8584856; doi:10.3390/ijms222112057)
Supplement: Supplementary file 1 [file ijms-22-12057-s001.zip › ijms-1399436-supplementary/Figure S6.pdf]

Online Resource 1

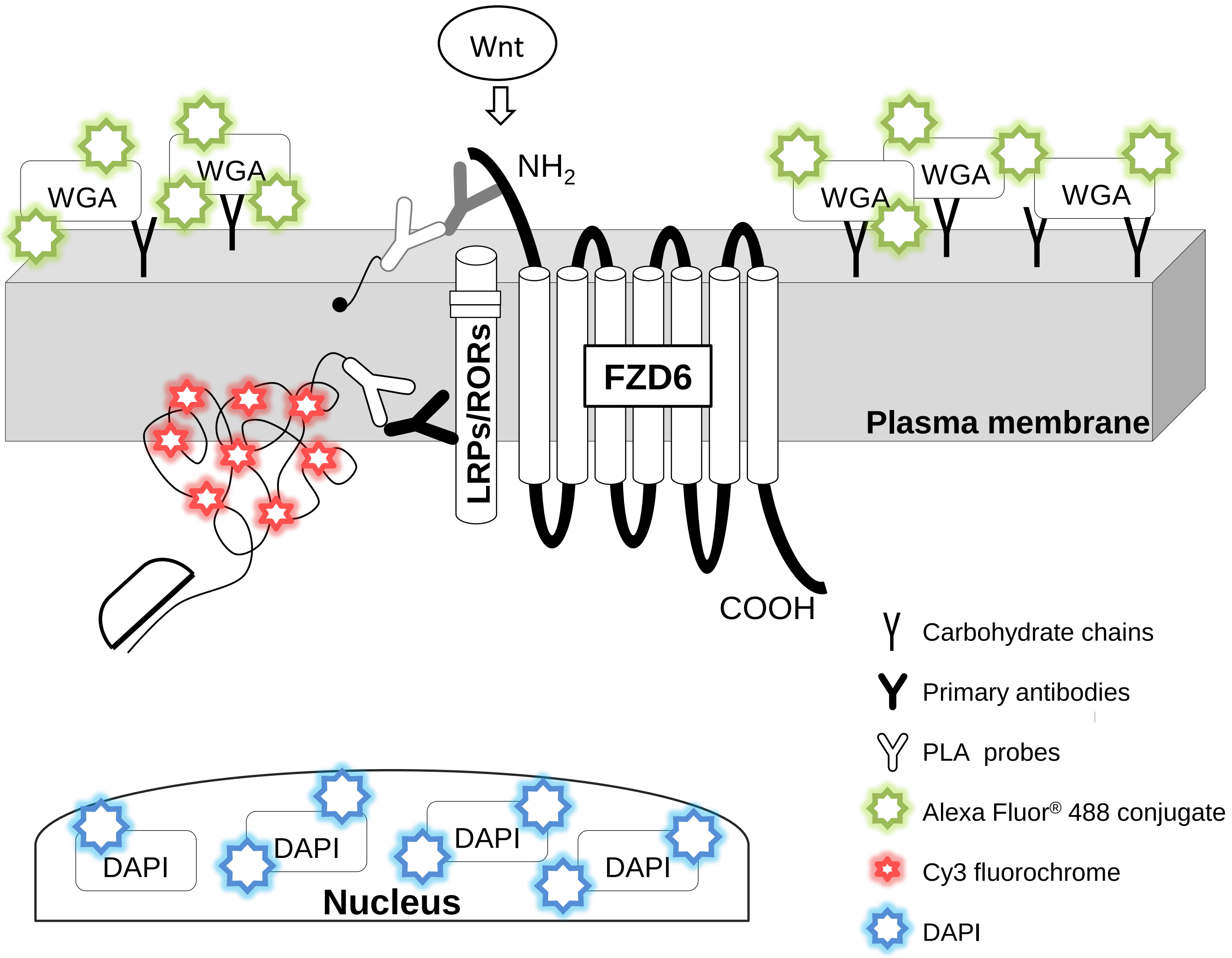

Figure S4: Schematic representation of triple-fluorescence staining. The PLA principle is based on close proximity (≤ 40 nm) of oligonucleotide-coupled secondary antibodies (PLA probes) that detect two different target proteins. Signal enhancement by a rolling circle amplification (RCA; 1000-fold) of a DNA circle and hybridization of Cy3 fluorophores allows visualization of FZD6-LRP/ROR interactions as red fluorescence signals. Alexa®488-coupled wheat germ agglutinin (WGA) binds to carbohydrate chains of plasma membranes and enabled labeling of cell surface (ROIs). Nuclei were stained with DAPI.
