# Supplementary material for: Immunocytochemical Analysis of Endogenous Frizzled-(Co-)Receptor Interactions and Rapid Wnt Pathway Activation in Mammalian Cells
Source: Int J Mol Sci. 2021 Nov 8;22(21):12057. doi: 10.3390/ijms222112057 (PMC8584856; doi:10.3390/ijms222112057)
Supplement: Supplementary file 1 [file ijms-22-12057-s001.zip › ijms-1399436-supplementary/Figure S7.pdf]

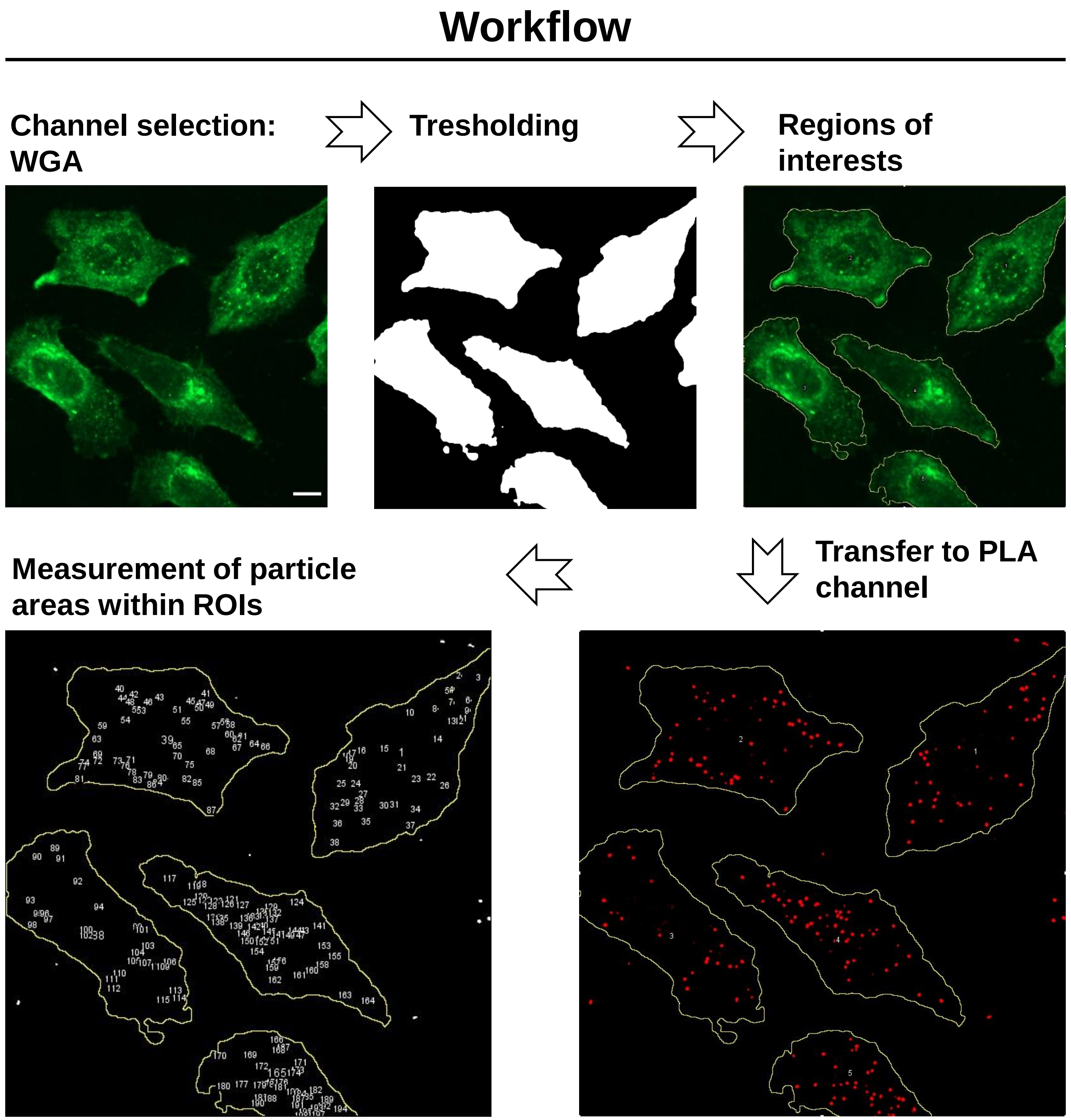

Figure S7: Workflow of particle analysis. Quantification of PLA signals at single cell level. Samples were analyzed using a LSM 800 confocal laserscanning microscope (Carl Zeiss, Jena, Germany). Images were acquired at constant settings; pinhole was appropriately adjusted to 47 µm to capture all PLA signals. After tresholding, single cells could be defined as regions of interests within the WGA channel (green: wheat germ agglutinin). Measurement of PLA signals was done within the original channel representing FZD6 interactions with co-receptors (red: FZD6-LRP6; FZD6-ROR1). The area of each PLA signal was determined. For quantification, sizes of PLA signals in single cells were totalled and normalized to the area of the cell. Approximately 200 single cells were analyzed per PLA and treatment condition. Scale bar: 10µm.
